# Trends in Cardiovascular Disease Risk Factors in People with and without Diabetes Mellitus: A Middle Eastern Cohort Study

**DOI:** 10.1371/journal.pone.0112639

**Published:** 2014-12-02

**Authors:** Younes Jahangiri-Noudeh, Samaneh Akbarpour, Mojtaba Lotfaliany, Neda Zafari, Davood Khalili, Maryam Tohidi, Mohammad Ali Mansournia, Fereidoun Azizi, Farzad Hadaegh

**Affiliations:** 1 Prevention of Metabolic Disorders Research Center, Research Institute for Endocrine Sciences (RIES), Shahid Beheshti University of Medical Sciences, Tehran, Islamic Republic of Iran; 2 Department of Epidemiology and Biostatistics, School of Public Health, Tehran University of Medical Sciences, Tehran, Islamic Republic of Iran; 3 Endocrine Research Center, Research Institute for Endocrine Sciences (RIES), Shahid Beheshti University of Medical Sciences, Tehran, Islamic Republic of Iran; Azienda Ospedaliero-Universitaria Careggi, Italy

## Abstract

**Aims/Hypothesis:**

To investigate secular trends in cardiovascular disease (CVD) risk factors during a decade of follow-up in a Middle Eastern cohort, and to compare observed trends between diabetic and non-diabetic populations.

**Methods:**

In a population of 6181 participants (2622 males and 3559 females), diabetes status and CVD risk factors were evaluated in 4 study phases from 1999–2011. 1045 subjects had type 2 diabetes mellitus at baseline and 5136 participants were diabetes-free. To examine the trends of CVD risk factors, generalized estimation equation models were constructed. The interaction between the diabetes status and each phase of the study was checked in a separate model.

**Results:**

During the follow-up period diabetic females significantly gained better control of their blood pressure, serum low density lipoprotein cholesterol and general and central obesity measures compared to non-diabetic counterparts, although 60% of them had high BP and 64% had high serum LDL-C levels till the end of the study. Diabetic males however, experienced significantly better control on their serum LDL-C and general and central obesity measures compared to their non-diabetic controls; but 24% of them were still smoker, 63% had high BP and 60% had high serum LDL-C levels at the end of the follow-up (all Ps _interaction_ <0.05). Use of lipid-lowering and antihypertensive medications increased consistently in both diabetic and non-diabetic populations.

**Conclusions/Interpretation:**

Although CVD risk factors have been controlled to some extent among diabetic population in Iran, still high numbers of people with diabetes have uncontrolled CVD risk factors that prompt more attention.

## Introduction

The prevalence of clinical type 2 diabetes mellitus is increasing rapidly in most regions of the world and the disease has emerged as a major public health problem worldwide [1], [2]. It is estimated that developing countries in Asia and the Middle East, will have the largest increases in the prevalence of type 2 diabetes mellitus by 2030, a rise related to a major shift in life style and nutrition in these countries [3], [4]. In a previous study, the annual age-standardized incidence rates of type 2 diabetes in Iranian population is reported to be 9.94 per 1000 person-years [5]. One of the most important complications of type 2 diabetes mellitus is cardiovascular disease (CVD) imposing heavy financial burdens to societies. [6]; In fact, diabetes itself approximately doubles the risk of developing CVD [7]. Other major risk factors of CVD both in diabetic and non-diabetic people are hypertension, hyperlipidemia, obesity and smoking [8]–[10].

Recent studies have demonstrated that the use of more aggressive targets for risk factor control among individuals with type 2 diabetes mellitus results in a reduced incidence of CVD events [11]–[15]. However, there are a few investigations showing substantial declines in dyslipidemia, blood pressure, and cigarette smoking in individuals with type 2 diabetes mellitus [16]–[18]. Although favorable secular trends in CVD risk factors have been illustrated in general population in most regions of the world [19]–[23], there are few studies comparing diabetic with non-diabetic populations [17], which makes it difficult to generalize the efficacy of preventive programs in people with diabetes worldwide. Moreover, investigations in the field of secular trends in traditional CVD risk factors are lacking, both in general population and diabetic subjects in Middle Eastern countries, despite a high prevalence and incidence of CVD risk factors [24]. The objective of the present study was to investigate secular trends of traditional CVD risk factors during a decade of follow-up of a Middle Eastern cohort, Tehran Lipid and Glucose Study (TLGS); we also aimed to compare the trends observed in diabetic and non-diabetic people.

## Methods

### Study Subjects

**Figure 1:**
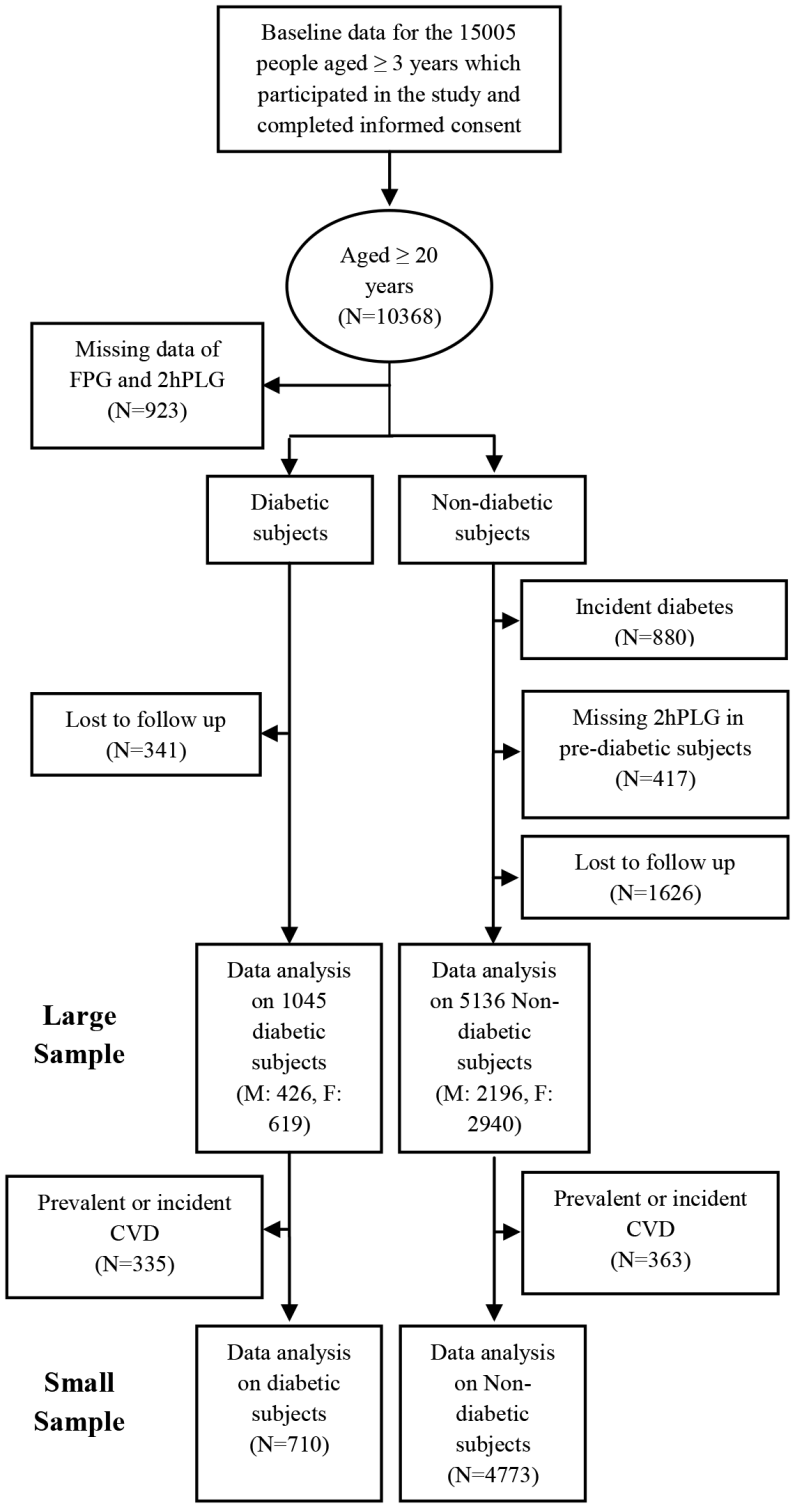
Flowchart demonstrating the selection of study participants; Teheran Lipid and Glucose Study (1999–2011). FPG, fasting plasma glucose; 2hPLG, 2-hour post-load plasma glucose; CVD, cardiovascular disease; M, male; F, female

Detailed descriptions of TLGS have been reported elsewhere [25]. Briefly, the TLGS is a community-based prospective study performed on a representative sample of residents of district 13 of Tehran, the capital city of Iran. Following recruitment of the subjects from the selected population and a baseline assessment (1999–2002) a total of 3 follow-up evaluations were done until December 2011 (a total of 4 study phases) with intervals about 3 years. Of 15005 individuals, aged ≧3 years enrolled in the first examination, those aged ≧20 years (n = 10368), were considered for the current study. The exclusion criteria consisted of missing baseline assessments for both fasting plasma glucose (FPG) and 2-hour post-load plasma glucose (2hPLG) (n = 923) and loss to follow-up after the baseline assessment (n = 1967). Among diabetes-free subjects at baseline, we excluded those who had a FPG ≧5.05 mmol/L with a missing 2hPLG (n = 417), while participants with missing data on 2hPLG at all follow up visits who had FPG levels below 5.05 mmol/L were considered diabetes-free [26]. Additionally, we excluded those with incident diabetes in any of the follow-up assessments (n = 880) (Figure 1).

Finally, the study population consisted of a total of 6181 participants (1045 diabetic and 5136 non-diabetic subjects) followed up until December 2011, with a median period of 9.56 (Inter-quartile range: 1.80) years. Initial analyses were conducted in the study population (large sample) and then repeated in subjects without prevalent or incident CVD event forming the small sample (Figure 1).

The study design was approved by the Institutional Review Board (IRB) of the Research Institute for Endocrine Sciences (RIES), Shahid Beheshti University of Medical Sciences, and all participants provided written informed consent.

### Clinical and anthropometric measurements

Subjects were interviewed by trained interviewers using pretested questionnaires. Information on age, sex, past medical history of CVD and high FPG, medication use and smoking habits were collected. Anthropometric measures including weight, height, hip and waist circumferences (WC) were recorded using standard protocols [25]. Body mass index (BMI) was calculated as weight in kilograms divided by height in square meters. Blood pressure (BP) was measured twice in a seated position after 15 min resting using a standard mercury sphygmomanometer (calibrated by Iranian Institute of Standards and Industrial Researches). Waist to hip ratio (WHR) was calculated by dividing WC by hip circumference.

### Laboratory measurements

After 12–14 hours of overnight fasting, a venous blood sample was drawn and centrifuged within 30–45 minutes of collection. All blood sampling was done between 7.00 and 9.00 A.M. and all measurements were completed on the day of sampling. Details of laboratory measurements including FPG, total cholesterol (TC), high density lipoprotein cholesterol (HDL-C) and triglycerides (TGs) were reported elsewhere [19], [25]. Non high density lipoprotein cholesterol (Non-HDL-C) was calculated by subtracting HDL-C from TC. Low density lipoprotein cholesterol (LDL-C) was calculated according to the modified Friedewald formula [27].

### Definition of terms

Type 2 diabetes mellitus was ascertained among participants who had FPG ≧7 mmol/L or 2hPLG ≧11.1 mmol/L or were on glucose lowering medication or gave positive answer to the question: “Have you ever had a high blood sugar?”

Lipid goals were defined as follows:

- Normal non-HDL-C: Serum non-HDL-C<3.37 mmol/L in diabetic subjects and serum non-HDL-C<4.14 mmol/L in non-diabetic subjects
- Normal LDL-C: Serum LDL-C<2.59 mmol/L in diabetic subjects and serum LDL-C<3.37 mmol/L in non-diabetic subjects
- Normal HDL-C: Serum HDL-C>1.29 mmol/L in women and HDL-C>1.04 mmol/L in men
- Normal triglycerides: Serum TGs<1.69 mmol/L in all subjects [28].

General obesity was defined in both genders by BMI≧30 kg/m^2^and central adiposity was defined as WC≧95 cm [29].

Participants who had a systolic blood pressure (SBP) ≧140 mmHg or a diastolic blood pressure (DBP) ≧90 mmHg (≧80 in diabetic subjects [30]) or were on antihypertensive drugs were referred to as hypertensive. Current smokers were defined as participants who were smoking cigarettes daily or occasionally as well as those who used water pipe or pipe.

Details of the collection of cardiovascular outcome data have been published elsewhere [25]. In the current study, cardiovascular disease outcome was defined as the first CVD event, including definite myocardial infarction (MI), probable MI, unstable angina, angiographic-proven coronary heart disease and stroke (as defined by a new neurological deficit that lasted more than 24 h).

### Statistical Analyses

The baseline characteristics are presented as mean (standard deviation) for numerical variables and number (percentage) for the categorical measures. The only numerical variable with skewed distribution was TGs for which median (interquartile range) was calculated. Differences in descriptive baseline characteristics were explored using Student's independent t-test between several couples of independent groups such as men and women, follow up and non-follow up, diabetic and non-diabetic populations. The Mann-Whitney U test (non-parametric equivalent to independent t-test) was applied to compare baseline TGs values. To assess the independence of two categorical variables chi square test or Fisher's exact test was used. To investigate the secular longitudinal trends of FPG, HDL-C, TG, TC, non-HDL-C, LDL-C, WC, WHR, SBP, DBP, smoking and BMI, generalized estimation equation (GEE) method was constructed. The GEE developed by Liang and Zeger, is a widely used estimation method for marginal (i.e., population-averaged) modeling of repeated data [31]. Models for examination of time trend were fitted separately for diabetic and non-diabetic groups and marginal (age-adjusted) means and P values for trend were reported in each group. The interaction between the diabetes status and each phase of the study was checked in a separate model; for this purpose, we entered the cross-product term (interaction term) in a single model including both diabetic and non-diabetic subjects. Furthermore, the interaction of type 2 diabetes status with each phase of study were also examined in those participants who reached the goal of lipid levels or blood pressure, consumed drugs for type 2 diabetes mellitus, hypertension or dyslipidemia as well as those who were considered obese with a similar method.

Because of significant difference in the subjects' age at the baseline visit between diabetic and non-diabetic groups, we adjusted all statistical models for the participants' baseline age to eradicate the potential confounding effect of age. We repeated the GEE analysis in the small sample (CVD-free) population, as well. All analyses were done, separately in females and males using STATA statistical software (version 12 SE).

## Results

### Baseline characteristics of the study population

As shown in Table 1, the diabetic participants showed a worse CVD risk profile than their non-diabetic counterparts at baseline, excluding the rate of cigarettes smoking in females and HDL-C levels in males. A gender-specific comparison of the included subjects and their lost-to-follow-up counterparts in diabetic and non-diabetic populations is shown in Table S1 in File S1. The mean CVD risk factor levels in each TLGS phase are presented in Tables S2 and S3 in File S1.

**Table 1:** Baseline characteristics of participants by gender and diabetes status^a^; Teheran Lipid and Glucose Study (March 1999- December 2011).

	Male (n = 2622)			Female (n = 3559)		
	Diabetic (n = 426)	Non-diabetic (n = 2196)	P value	Diabetic (n = 619)	Non-diabetic (n = 2940)	P value
Age (years)	56.00(12.05)	42.08(14.18)	P<0.001	53.06(10.78)	38.92(12.29)	P<0.001
FPG (mmol/L)	8.15(3.17)	4.97(0.47)	P<0.001	8.82(3.73)	4.85(0.47)	P<0.001
HDL-C (mmol/L)	0.98(0.25)	0.99(0.23)	0.197	1.12(0.27)	1.18(0.29)	P<0.001
TGs (mmol/L)^b^	2.86(2.37)	1.94(1.21)	P<0.001	2.71(1.77)	1.61(0.95)	P<0.001
TC (mmol/L)	5.76(1.18)	5.23(1.08)	P<0.001	6.29(1.33)	5.33(1.15)	P<0.001
Non-HDL-C (mmol/L)	4.76(1.16)	4.23(1.08)	P<0.001	5.16(1.31)	4.14(1.16)	P<0.001
LDL-C (mmol/L)	3.64(0.92)	3.36(0.87)	P<0.001	4.03(1.04)	3.36(0.94)	P<0.001
WC (cm)	94.87(10.25)	87.42(10.89)	P<0.001	96.51(11.33)	85.53(11.95)	P<0.001
SBP (mmHg)	132.06(22.39)	118.49(16.71)	P<0.001	134.15(22.35)	114.92(16.50)	P<0.001
DBP (mmHg)	81.48(12.03)	76.89(10.51)	P<0.001	83.25(10.97)	76.27(10.05)	P<0.001
BMI (kg/m^2^)	27.56(3.83)	25.48(3.93)	P<0.001	29.75(5.06)	26.97(4.67)	P<0.001
Smoking, No. (%)	97(23.5)	616(28.2)	0.049	23(3.7)	102(3.5)	0.746

a values are presented as mean (SD) unless otherwise indicated

b presented as median (interquartile range).

FPG, fasting plasma glucose; HDL-C, high-density lipoprotein cholesterol; TGs, triglycerides; TC, total cholesterol; Non-HDL-C, non-high-density lipoprotein cholesterol; LDL-C, low-density lipoprotein cholesterol; WC, waist circumference; SBP, systolic blood pressure; DBP, diastolic blood pressure; BMI, body mass index.

### Changes in the mean levels of CVD risk factors

**Figure 2:**
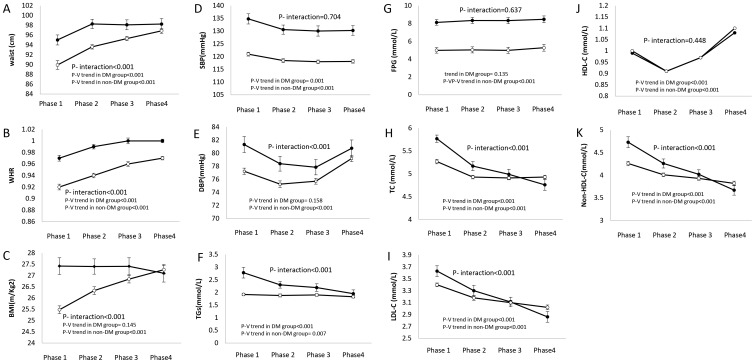
Age-adjusted mean levels of cardiovascular risk factors in each phase in male subjects. Mean levels of cardiovascular risk factors of diabetic and non-diabetic participants was calculated separately with adjustment of related participants' age in each phase while the interaction of diabetes status was assessed in a pooled model consisting both diabetic and non- diabetic participants. Black circles  =  diabetic group; white circles  =  non diabetic group; DM, diabetes mellitus; WHR, waist to hip ratio; BMI, body mass index; SBP, systolic blood pressure; DBP, diastolic blood pressure; TGs, triglycerides; FPG, fasting plasma glucose; TC, total cholesterol; LDL, low-density lipoprotein cholesterol; HDL-C, high-density lipoprotein cholesterol; Non-HDL-C, non-high-density lipoprotein cholesterol.

**Figure 3:**
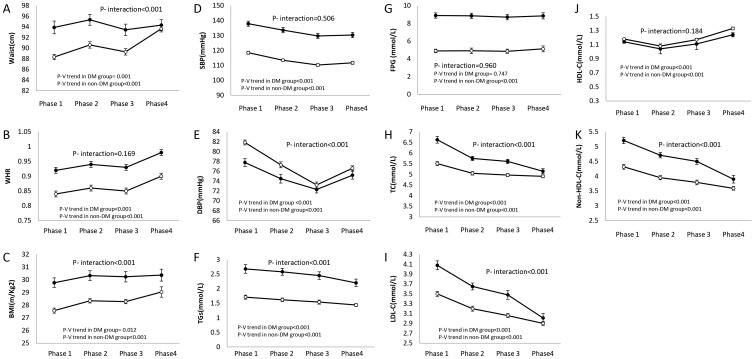
Age-adjusted mean levels of cardiovascular risk factors in each phase in female subjects. Mean levels of cardiovascular risk factors of diabetic and non-diabetic participants was calculated separately with adjustment of related participants' age in each phase while the interaction of diabetes status was assessed in a pooled model consisting both diabetic and non-diabetic participants. Black circles  =  diabetic group; white circles  =  non diabetic group; DM, diabetes mellitus; WHR, waist to hip ratio; BMI, body mass index; SBP, systolic blood pressure; DBP, diastolic blood pressure; TGs, triglycerides; FPG, fasting plasma glucose; TC, total cholesterol; LDL, low-density lipoprotein cholesterol; HDL-C, high-density lipoprotein cholesterol; Non-HDL-C, non-high-density lipoprotein cholesterol.

Figures 2 and 3 illustrate age-adjusted trends of CVD risk factors in males and females, respectively. As of comparing the trend for anthropometric measures, BMI demonstrated a non-significant decrease in diabetic males (P = 0.145) compared to their non-diabetic controls who gained a significant average value over time (mean BMI = 25.48 kg/m^2^ in phase 1, vs. mean BMI = 27.26 kg/m^2^ in phase 4, P _trend_ <0.001); in female participants however both diabetic (mean BMI = 29.77 kg/m^2^in phase 1 vs. mean BMI = 30.37 kg/m^2^ in phase 4, P _trend_ = 0.012) and non-diabetic (mean BMI = 27.57 kg/m^2^in phase 1 vs. mean BMI = 29.04 kg/m^2^ in phase 4, P _trend_<0.001) subjects had a statistically significant rise in their BMI. The changes of BMI in diabetic subjects were significantly different form their non-diabetic peers (P _interaction_<0.001 for both genders). In male participants, WC and WHR significantly increased regardless of the subjects' baseline diabetes status but were more pronounced in non-diabetic people. Meanwhile, the trend in the female population was different as diabetic participants showed a significant decline in their WC (P _trend_ = 0.001), but their non-diabetic peers experienced a statistically significant incremental trend (P _trend_ <0.001). However, after adjusting for their hip circumference, by calculating WHR, both diabetic and non-diabetic women showed an incremental trend over time but no statistically significant interaction was observed with their baseline diabetes status (P _interaction_ = 0.169). Although SBP decreased significantly over a decade in both diabetic (mean SBP = 134.81 and 137.92 mmHg in phase 1 vs. mean SBP = 130.27 and 130.32 mmHg in phase 4 for males and females, respectively) and non-diabetic groups (mean SBP = 120.97 and 118.51 mmHg in phase 1 vs. mean SBP = 118.12 and 111.74 mmHg in phase 4 for males and females, respectively), it was in fact independent of the subjects baseline diabetes status (P _interaction_  = 0.704 and 0.506 for males and females, respectively). Diastolic blood pressure however, had more complicated trends as diabetic males experienced a non-significant decrease (P _trend_ = 0.158), while their non-diabetic peers showed generally a statistically significant decrease in their DBP (P _trend_ <0.001). Among female participants however, both diabetic and non-diabetic individuals experienced generally statistically significant decreases in DBP during the follow-up period with the diabetic group having higher decrease (P _interaction_ <0.001).

Over a decade, diabetic individuals serum TC, TGs, LDL-C and non HDL-C decreased with a further acceleration, compared to non-diabetic controls in both genders. Serum HDL-C levels increased significantly in both diabetic and non-diabetic population without any significant differences according to the baseline diabetes status. Fasting plasma glucose levels showed a non-significant trend in diabetic males (P _trend_ = 0.135) and females (P _trend_ = 0.747), although non-diabetic controls experienced statistically significant incremental trends (P _trend_ <0.001 for both genders).

Trends in the rates of reaching target values of lipid levels and blood pressure as well as rate of general and central obesity, cigarette smoking and medications use during the follow-up is shown in Table 2. The rate of general and central obesity increased significantly in the non-diabetic population. The prevalence of BMI ≧30 kg/m^2^ stayed relatively stable in diabetic subjects of both genders (P _trend_ = 0.98 and 0.05 for males and females respectively), however, this prevalence increased significantly over time in the non-diabetic population of both genders (2.17 and 1.29 fold increases in males and females, respectively, comparing last follow up versus baseline data). Ratios of abdominal adiposity also showed a similar dramatic increase in non-diabetic population (2.21 and 1.38 fold increases in males and females, respectively, comparing last follow up versus baseline data), compared to diabetic participants of both genders (1.26 and 1.15 fold increase in males and females, respectively, comparing last follow up versus baseline data). Although males did not have statistically significant changes in the BP control rates over this decade in either the diabetic or non-diabetic group (P _trend_  = 0.942 and 0.274, respectively, comparing last follow up versus baseline data); female participants demonstrated favorable rates of BP control (1.46 and 1.05 fold increases in the rate of BP control in diabetic and non-diabetic subjects respectively, P _interaction_<0.001). In both genders the percentage of the subjects who met the target normal levels of serum lipids increased over time in both diabetic and non-diabetic groups with the diabetic individuals having more prominent increase in reaching the goal of serum lipids, excluding HDL-C in both genders. The prevalence of cigarette smoking increased significantly among diabetic and non-diabetic participants both in males and females (not significant in diabetic males, P _trend_ = 0.107). The use of lipid-lowering and antihypertensive drugs increased consistently in both diabetic and non-diabetic males and females (excluding antihypertensive drug use in non-diabetic females), the increases which was not modified by the baseline diabetes status.Finally, in diabetic subjects the consumption of anti-diabetic medication significantly increased about 2-fold in males (51.51% in phase 4 vs. 24.15% in phase 1) and 1.7-fold in females (66.70% in phase 4 vs. 39.73% in phase 1).

**Table 2:** Age-adjusted percentage of subjects in diabetic and non-diabetic groups who reached goal levels of lipid measures and blood pressure and trends of obesity and medications consumption; Teheran Lipid and Glucose Study (March 1999- December 2011).

Males		Phase1 (1999–2002)	Phase2 (2002–2005)	Phase3 (2005–2008)	Phase4 (2008–2011)	P_trend_	P_interaction_
Reached HDL-C goal (%)	DM	35.91	23.48	30.92	50.45	P<0.001	0.91
	Non-DM	37.94	23.01	31.64	52.98	P<0.001	
Reached TGs goal (%)	DM	28.77	37.65	43.37	49.40	P<0.001	P<0.001
	Non-DM	51.20	54.15	53.00	55.16	0.01	
Reached Non-HDL-C goal (%)	DM	9.91	17.12	25.90	39.21	P<0.001	P<0.001
	Non-DM	47.76	56.44	60.38	64.05	P<0.001	
Reached LDL-C goal (%)	DM	10.34	18.45	26.79	40.36	P<0.001	P<0.001
	Non-DM	50.40	61.19	65.92	68.42	P<0.001	
Reached blood pressure control goal (%)	DM	38.85	47.39	47.49	37.36	0.942	0.036
	Non-DM	81.23	85.38	84.86	80.25	0.274	
BMI> = 30 kg/m2	DM	25.53	25.52	26.24	25.31	0.980	P<0.001
	Non-DM	11.23	19.00	20.88	24.48	P<0.001	
WC> = 95 cm	DM	54.82	70.03	67.44	69.26	P<0.001	P<0.001
	Non-DM	27.52	50.39	56.55	61.08	P<0.001	
Smoking (%)	DM	20.18	23.42	22.69	24.23	0.107	P<0.001
	Non-DM	25.54	34.8	34.02	35.43	P<0.001	
Glucose-lowering medication use (%)	DM	24.15	37.25	48.08	51.51	P<0.001	-
	Non-DM	-	-	-	-	-	
Lipid-lowering medication use (%)	DM	5.71	9.42	13.63	23.52	P<0.001	0.297
	Non-DM	1.59	1.98	2.68	6.00	P<0.001	
Antihypertensive medication use (%)	DM	19.17	20.45	10.58	30.18	0.012	0.266
	Non-DM	4.59	4.91	3.21	7.21	0.002	
Females							
Reached HDL-C goal (%)	DM	21.82	15.33	22.07	37.50	P<0.001	0.144
	Non-DM	29.36	17.18	27.56	48.58	P<0.001	
Reached TGs goal (%)	DM	25.50	26.65	30.33	41.93	P<0.001	P<0.001
	Non-DM	58.57	63.85	66.87	72.06	P<0.001	
Reached Non-HDL-C goal (%)	DM	5.65	10.25	14.65	35.83	P<0.001	P<0.001
	Non-DM	46.26	59.66	67.17	72.87	P<0.001	
Reached LDL-C goal (%)	DM	6.29	11.04	15.43	36.32	P<0.001	P<0.001
	Non-DM	47.14	61.64	68.30	73.60	P<0.001	
Reached blood pressure control goal (%)	DM	27.67	43.59	51.03	40.43	P<0.001	P<0.001
	Non-DM	82.94	88.92	90.97	87.58	P<0.001	
BMI> = 30 kg/m2	DM	46.86	53.22	50.33	53.60	0.05	P<0.001
	Non-DM	28.78	38.28	34.57	37.37	P<0.001	
WC> = 95 cm	DM	60.13	68.43	62.99	69.28	0.005	P<0.001
	Non-DM	30.72	40.12	34.56	42.51	P<0.001	
Smoking (%)	DM	3.17	6.56	4.67	4.95	P<0.001	0.005
	Non-DM	3.39	5.49	5.6	6.82	P<0.001	-
Glucose-lowering medication use (%)	DM	39.73	47.59	58.48	66.70	P<0.001	P<0.001
	Non-DM	-	-	-	-		
Lipid-lowering medication use (%)	DM	13.35	14.47	22.11	35.02	P<0.001	0.714
	Non-DM	2.10	2.12	3.39	5.00	P<0.001	
Antihypertensive medication use (%)	DM	31.66	32.55	19.05	41.91	0.038	0.203
	Non-DM	8.01	8.22	4.26	8.60	0.77	

HDL-C, high-density lipoprotein cholesterol; TGs, triglycerides; Non-HDL-C, non-high-density lipoprotein cholesterol; LDL-C, low-density lipoprotein cholesterol; BMI, body mass index; WC, waist circumference.

The results remained unchanged after exclusion of the subjects with prevalent or incident CVD event in any of study phases (Tables S4, S5 in File S1).

## Discussion

In the present study of a Middle Eastern population, during a decade of follow-up, general and central obesity measures remained more stable in people with type 2 diabetes mellitus, compared to the non-diabetic population, which experienced dramatic increases. Also serum lipid profile control increased well over a decade in both diabetic and non-diabetic participants of both genders; the diabetic population, however, generally experienced higher rates of improvement in their serum lipid profiles, compared to their non-diabetic controls. These results were consistent with the increase in the usage of lipid-lowering medications in all subgroups, although about 60% of diabetic population did not reach the therapeutic goals of non-HDL-C and LDL-C levels till 2011. Control of hypertension was more successful in females, among whom diabetic subjects experienced the highest rate of BP control over time; among males however, there was no significant change in the control of hypertension either in diabetic subjects or in their non-diabetic peers. Despite the significant increase of consumption of anti-hypertensive medications in both groups, about 60% of both males and females with diabetes still had high BPs at the end of follow-up period. In both genders, we did not find improvement in smoking control among diabetic patients and cigarette smoking actually increased among the non-diabetic group.

To the best of our knowledge, our study is the first to show the trends of CVD risk factors in people with type 2 diabetes mellitus vs. non-diabetic population in a Middle Eastern cohort. Although mostly consistent with the results of the studies from the other parts of the world, our results were in contrast in some aspects. There are few studies regarding trends of CVD risk factors in diabetic populations [10], [16], [17], [32].

Researchers from the Framingham Heart Study showed that, diabetic people compared to their non-diabetic counterparts, experienced higher amounts of increase in their BMI as well as decreases in their serum LDL-C levels and also a similar magnitude of decrease in their SBPs [10]; they emphasized that the diabetic population had not met the necessary declines in their CVD risk factors to overcome their increased risk of CVD. Two other studies from the United States [16], [18] using the National Health and Nutrition Examination Survey (NHANES) data showed a dramatic decrease in the mean values of TC, SBP, DBP and smoking rates among a diabetic population over a 30-year period (1971–2000). However, at the end of this period, one of two people with diabetes still had high TC, one of three had high BP and one of six was a smoker. Also a declining trend in the mean values of SBP, HbA1C, TC and TC/HDL-C ratio, but no significant linear trend for current smoking status, from 1999 to 2008 was observed among US adults with diagnosed type 2 diabetes mellitus. Another study from England reported substantial reductions in SBP, DBP and TC levels and increase in BMI of people with type 2 diabetes mellitus. [17].

Results from national studies published by World Health Organization [33] have been illustrated the increasing value of BMI in both genders among Iranian population, during 1999–2009, without considering the diabetes status of the participants. The stability or decreases in the diabetic subjects' BMI observed in our study were in contrast with a previous report of increasing trends in BMI of diabetic adults of the United States [32]. Our observation might be explained by poor control of the disease in people with diabetes in our cohort, as diabetes itself can cause decreases in body weight and BMI [34]. The increase in the WHR despite stable or decreasing BMI, seen especially among female diabetic subjects in our study could be attributed to an increase in the ratio of the population with normal weight obesity. This type of obesity especially in women has independently been associated with an increased risk of cardiovascular mortality [35]. A recent global study after pooled analysis of 97 prospective cohorts showed that the excess risk of BMI, overweight and obesity could mainly be explained by three metabolic mediators. They suggested that interventions that reduce high blood pressure, cholesterol and glucose might address about half of excess risk of coronary heart disease and three-quarters of excess risk of stroke associated with high BMI [36].

We have previously shown that the population-attributable hazard fraction (PAHF) of hypertension for CVD events and CVD-related mortality among diabetic population was calculated to be 29.6% and 27.9%, respectively [37], emphasizing strongly the importance of controlling hypertension especially among diabetic populations. In the present study, 63% of diabetic males and 60% of diabetic females still have uncontrolled blood pressure levels, numbers that are almost double that of a previous study from the United States [16]. If left untreated, this may cause a high incidence of cardiovascular complications in the future.

Plasma glucose level is one of the metabolic mediators that showed an upward trend consistent with the increasing prevalence of general and central obesity especially among non-diabetic population in our study. The growing trend in age-adjusted FPG values in our population regardless of the subjects' diabetes status also was reported previously elsewhere [19]. This developing trend can be attributed to increasing sedentary life style among Iranian population. Given the strong predictability of current FPG measures in future hazards of developing diabetes [5], the rising levels of FPG over time could be an alarming sign for much higher incidences of diabetes among Iranian population in future years.

Hypercholesterolemia is also still very common in the current study despite significantly increased control over the last decade. Furthermore, in line with the current study and our recent findings[19], cross-sectional National studies conducted by Ministry of Health and Medical Education among Iranian adult population in whole country, showed significant decrease in level of high total cholesterol (Etemad K., Center for Noncommunicable Diseases Control, Ministry of Health and Medical Education, Tehran, Iran). Moreover, our observation of decreasing trends in the prevalence of hypercholesterolemia among diabetic population is consistent with previous reports [10], [16]–[18], [38]. Increase in the control of hypercholesterolemia can be attributed to significantly increased utilization of lipid-lowering medications among which statins play a pivotal role. Nevertheless, in our study still 2 out of 3 people with diabetes had uncontrolled serum LDL-C levels; also one in 2 diabetic males and 2 out of 3 diabetic females did not reach their desired serum levels of HDL-C, these results highlight a higher CVD risk in diabetic population, especially among females, a warning that needs healthcare professionals and policymakers' attention. We used the traditionally recommended cutoff values of serum LDL-C levels of <2.59 mmol/L [30] to classify our population. However, the new cholesterol treatment guidelines emphasize on lowering serum LDL-C levels in all diabetic individuals, aged 40–75 years and those with LDL-C level between 1.81–4.90 mmol/L [39]. Although lipid-lowering drugs use increased in our population of diabetic participants over a 10-year period, at the end of our follow-up period, only one-fifth of diabetic males and one-third of diabetic females were taking lipid-lowering medications including statins. In a comparison to the new treatment guidelines [39] our diabetic population are still obviously under-treated for their serum cholesterol levels.

Cigarette smoking is another important risk factor that showed a dramatic increase among populations of both genders. This is inconsistent with previous reports of the other populations [10], [16]-[18]. We previously have shown that even smoking 10 cigarettes a day or being a past smoker almost doubled the risk of CVD events during a 9.5 year follow-up [40]. In the present study, although the rate of smoking among females remains much lower than that of other studies, its 10-year trend shows a 56% increase among diabetic females, while non-diabetic females actually had a 2-fold increase in their smoking rates. Among males also smoking rates increased 20% and 39% from their baseline smoking rates in diabetic and non-diabetic subjects, respectively. Smoking by itself have been shown to increase risk of type 2 diabetes among overweight men (HR = 1.33) with an attributable proportion due to an interaction between overweight and heavy smoking being 40% [41]. The same study demonstrated that smoking reduces the risk of autoimmune diabetes dose-dependently possibly because of inhibitory effects on the autoimmune processes. If neglected, increases in the rates of cigarette smoking in our study population places both diabetic and non-diabetic populations at dramatically increased risk of future CVD [40]. In a recent study, it has been proposed that the five most important preventive measures in diabetes by the order of importance are smoking cessation, BP control, metformin therapy, lipid reduction and glycemic control. The authors have suggested that approach to the treatment of diabetic patients requires a shift in the thinking of patients and physicians i.e. putting more emphasis on other CVD risk factors including cigarette smoking and hypertension and hypercholesterolemia control rather than tight glucose control [42].

As the strengths of our study, we used data of an ongoing cohort study to assess the trends overtime. A relatively long period of follow-up in addition to using standardized measurement methods by educated health professionals rather than self-reported measures increases both the reliability and accuracy of our data.

As for limitations, our study actually shows an optimistic picture of CVD risk control among diabetic and non-diabetic populations since inclusion of subjects in an ongoing study can increase the level of attention they pay to controlling their health risks (cohort effect). Therefore, the burden of measured risk factors will be much higher in the context of the community. Moreover our study is subject to the survival bias as the subjects with very high levels of risk factors could be lost in successive phases due to death from CVD events or other complications. However, our results remained essentially unchanged, when we excluded prevalent or incident cases of CVD. In addition, data regarding inflammatory markers and mediators were lacking in our study and we could not assess the effects of changes in levels of these factors which have been shown to be of prime relevance in relation to diverse chronic diseases in Middle-Eastern population [43].

In conclusion, this study shows that among CVD risk factors hypercholesterolemia caught the most attention of the healthcare professionals in Iran, while other risk factors including hypertension, central obesity and cigarette smoking are substantially neglected. Also, despite dramatically increased usage of lipid-lowering drugs, a high percentage of the population is still beyond the control limits. Therefore, higher efforts by healthcare workers and policymakers are vital to prevent increased incidence of CVD events and the resulting mortality, both in diabetic and non-diabetic populations in Iran in the future.

## Supporting Information

File S1 **Supporting tables**. Table S1, Baseline characteristics of the participants with and without follow-up in both diabetic and non-diabetic groups^a^; Teheran Lipid and Glucose Study (1999–2011) (^a^ values are presented as mean (SD) unless otherwise indicated; ^b^ P value for differences between followed vs. not followed subjects; ^c^ P value for differences between followed diabetic vs. followed non-diabetic subjects; ^d^formula for calculating LDL-C values as follows: LDL-C (mg/dl)  =  Non-HDL-C×90% - TG ×10%; FPG, fasting plasma glucose; HDL-C, high-density lipoprotein cholesterol; TGs, triglycerides; TC, total cholesterol; Non-HDL-C, non-high-density lipoprotein cholesterol; LDL-C, low-density lipoprotein cholesterol; WC, waist circumference; SBP, systolic blood pressure; DBP, diastolic blood pressure; BMI, body mass index). Table S2, Average measures of CVD risk factors in diabetic and non-diabetic male participants in each phase of Teheran Lipid and Glucose Study (1999-2011) (^a^ formula for calculating LDL-C values as follows: LDL-C (mg/dl)  =  Non-HDL-C×90% - TG ×10%; FPG, fasting plasma glucose; HDL-C, high-density lipoprotein cholesterol; TGs, triglycerides; TC, total cholesterol; Non-HDL-C, non-high-density lipoprotein cholesterol; LDL-C, low-density lipoprotein cholesterol; WC, waist circumference; WHR, waist to hip ratio; SBP, systolic blood pressure; DBP, diastolic blood pressure; BMI, body mass index; CI, confidence interval). Table S3, Average measures of CVD risk factors in diabetic and non-diabetic female participants in each phase of Teheran Lipid and Glucose Study (1999–2011) (^a^formula for calculating LDL-C values as follows: LDL-C (mg/dl)  =  Non-HDL-C×90% - TG ×10%; FPG, fasting plasma glucose; HDL-C, high-density lipoprotein cholesterol; TGs, triglycerides; TC, total cholesterol; Non-HDL-C, non-high-density lipoprotein cholesterol; LDL-C, low-density lipoprotein cholesterol; WC, waist circumference; WHR, waist to hip ratio; SBP, systolic blood pressure; DBP, diastolic blood pressure; BMI, body mass index; CI, confidence interval). Table S4, Age- and sex-adjusted trends of CVD risk factors in diabetic and non-diabetic populations with no CVD events in each phase of Teheran Lipid and Glucose Study^a^ (1999–2011) (^a^Mean levels of cardiovascular risk factors of diabetic and non-diabetic participants was calculated separately with adjustment of related participants age and sex in each phase while the interaction of diabetes status was assessed in a pooled model consisting both diabetic and non-diabetic participants, using Generalized Estimation Equation method; ^b^formula for calculating LDL-C values as follows: LDL-C (mg/dl)  =  Non-HDL-C×90% - TG ×10%; FPG, fasting plasma glucose; HDL-C, high-density lipoprotein cholesterol; TGs, triglycerides; TC, total cholesterol; Non-HDL-C, non-high-density lipoprotein cholesterol; LDL-C, low-density lipoprotein cholesterol; WC, waist circumference; WHR, waist to hip ratio; SBP, systolic blood pressure; DBP, diastolic blood pressure; BMI, body mass index; CI, confidence interval). Table S5, The percentage of subjects in diabetic and non-diabetic groups without CVD event in any of study phases who reached goal levels of lipid measures and blood pressure and trends of obesity and different medications consumption^a^; Teheran Lipid and Glucose Study (1999–2011) (^a^The percentage of cardiovascular risk factors of diabetic and non-diabetic participants was calculated separately with adjustment of related participants' age in each phase while the interaction of diabetes status was assessed in a pooled model consisting both diabetic and non-diabetic participants, using Generalized Estimation Equation method. Goal of hypertension is defined as <140/90 mmHg for individuals without diabetes and <140/80 mmHg for individuals with diabetes. Goal of LDL-C is defined as <3.37 mmol/L for individuals without diabetes and <2.59 mmol/L for individuals with diabetes. Goal of non-HDL-C is defined as <4.14 mmol/L for individuals without diabetes and <3.37 mmol/L for individuals with diabetes. Goal of HDL-C is defined as >1.04 mmol/L for men and >1.29 mmol/L for women in both diabetic and non-diabetic population. Goal of Triglycerides is defined as <1.69 mmol/Lin both diabetic and non-diabetic population. General obesity is defined as a BMI of ≧30 kg/m^2^.Abdominal obesity is defined as, WC ≧95 cm in both genders; ^b^formula for calculating LDL-C values as follows: LDL-C (mg/dl)  =  Non-HDL-C×90% - TG ×10%; HDL-C, high-density lipoprotein cholesterol; TGs, triglycerides; Non-HDL-C, non-high-density lipoprotein cholesterol; LDL-C, low-density lipoprotein cholesterol; BMI, body mass index; WC, waist circumference).
(DOC)
