# Supplementary material for: Trends in Cardiovascular Disease Risk Factors in People with and without Diabetes Mellitus: A Middle Eastern Cohort Study
Source: PLoS One. 2014 Dec 2;9(12):e112639. doi: 10.1371/journal.pone.0112639 (PMC4251920; doi:10.1371/journal.pone.0112639)
Supplement: File S1 — Supporting tables. Table S1, Baseline characteristics of the participants with and without follow-up in both diabetic and non-diabetic groupsa; Teheran Lipid and Glucose Study (1999–2011) (a values are presented as mean (SD) unless otherwise indicated; b P value for differences between followed vs. not followed subjects; c P value for differences between followed diabetic vs. followed non-diabetic subjects; dformula for calculating LDL-C values as follows: LDL-C (mg/dl) = Non-HDL-C×90% - TG ×10%; FPG, fasting plasma glucose; HDL-C, high-density lipoprotein cholesterol; TGs, triglycerides; TC, total cholesterol; Non-HDL-C, non-high-density lipoprotein cholesterol; LDL-C, low-density lipoprotein cholesterol; WC, waist circumference; SBP, systolic blood pressure; DBP, diastolic blood pressure; BMI, body mass index). Table S2, Average measures of CVD risk factors in diabetic and non-diabetic male participants in each phase of Teheran Lipid and Glucose Study (1999-2011) (a formula for calculating LDL-C values as follows: LDL-C (mg/dl) = Non-HDL-C×90% - TG ×10%; FPG, fasting plasma glucose; HDL-C, high-density lipoprotein cholesterol; TGs, triglycerides; TC, total cholesterol; Non-HDL-C, non-high-density lipoprotein cholesterol; LDL-C, low-density lipoprotein cholesterol; WC, waist circumference; WHR, waist to hip ratio; SBP, systolic blood pressure; DBP, diastolic blood pressure; BMI, body mass index; CI, confidence interval). Table S3, Average measures of CVD risk factors in diabetic and non-diabetic female participants in each phase of Teheran Lipid and Glucose Study (1999–2011) (aformula for calculating LDL-C values as follows: LDL-C (mg/dl) = Non-HDL-C×90% - TG ×10%; FPG, fasting plasma glucose; HDL-C, high-density lipoprotein cholesterol; TGs, triglycerides; TC, total cholesterol; Non-HDL-C, non-high-density lipoprotein cholesterol; LDL-C, low-density lipoprotein cholesterol; WC, waist circumference; WHR, waist to hip ratio; SBP, systolic blood pressure; DBP, diasto [file pone.0112639.s001.doc]

|  | Diabetic Subjects | | | Non Diabetic Subjects | | |  |
| --- | --- | --- | --- | --- | --- | --- | --- |
|  | Followed  (n=1045) | Not Followed  (n=341) | P^b^ | Followed  (n=5136) | Not Followed  (n=1626) | P^b^ | P^c^ |
| *Males* |  |  |  |  |  |  |  |
| Age (year) | 56.09(12.03) | 60.16(13.45) | 0.001 | 42.08(14.18) | 42.14(15.81) | 0.940 | P<0.001 |
| FPG (mmol/L) | 8.15(3.18) | 8.81(3.46) | 0.031 | 4.97(0.47) | 4.99(0.53) | 0.324 | P<0.001 |
| HDL-C (mmol/L) | 0.98(0.25) | 0.98(0.29) | 0.99 | 0.99(0.23) | 0.98(0.24) | 0.410 | 0.198 |
| TGs (mmol/L), median (IQR) | 2.26(1.65) | 2.03(1.37) | 0.014 | 1.68(1.28) | 1.53(1.13) | 0.018 | P<0.001 |
| TC (mmol/L) | 5.73(1.14) | 5.70(1.13) | 0.479 | 5.23(1.08) | 5.14(1.06) | 0.051 | P<0.001 |
| Non-HDL-C (mmol/L) | 4.75(1.15) | 4.71(1.12) | 0.582 | 4.23(1.09) | 4.15(1.07) | 0.066 | P<0.001 |
| LDL-C (mmol/L)^d^ | 3.64(0.93) | 3.68(0.95) | 0.692 | 3.36(0.87) | 3.31(0.86) | 0.14 | P<0.001 |
| WC (cm) | 94.84(10.27) | 93.27(10.45) | 0.092 | 87.41(10.83) | 87.28(11.43) | 0.803 | P<0.001 |
| SBP (mmHg) | 132.01(22.40) | 135.11(24.49) | 0.145 | 118.49(16.71) | 119.89(19.37) | 0.064 | P<0.001 |
| DBP (mmHg) | 81.48(12.04) | 81.64(12.41) | 0.887 | 76.90(10.51) | 77.14(11.58) | 0.551 | P<0.001 |
| BMI (kg/m^2^) | 27.56(3.83) | 26.86(4.01) | 0.056 | 25.48(3.90) | 25.46(4.19) | 0.923 | P<0.001 |
| Smoking, No.(%) | 97(22.8) | 38(24.1) | 0.797 | 616(28.1) | 235(34.8) | 0.001 | 0.048 |
| *Females* |  |  |  |  |  |  |  |
| Age (year) | 53.05(10.78) | 57.95(11.28) | P<0.001 | 38.91(12.27) | 40.22(14.98) | 0.007 | P<0.001 |
| FPG (mmol/L) | 8.82(3.74) | 8.77(3.77) | 0.90 | 4.85(0.47) | 4.94(0.54) | P<0.001 | P<0.001 |
| HDL-C (mmol/L) | 1.12(0.27) | 1.14(0.31) | 0.555 | 1.18(0.29) | 1.17(0.29) | 0.246 | P<0.001 |
| TGs (mmol/L), median (IQR) | 2.35(1.58) | 2.76(1.70) | 0.726 | 1.68(1.28) | 1.53(1.13) | 0.098 | P<0.001 |
| TC (mmol/L) | 6.30(1.33) | 6.33(1.41) | 0.756 | 5.33(1.15) | 5.40(1.25) | 0.114 | P<0.001 |
| Non-HDL-C (mmol/L) | 5.17(1.31) | 5.18(1.39) | 0.834 | 4.14(1.16) | 4.23(1.25) | 0.063 | P<0.001 |
| LDL-C (mmol/L)^d^ | 4.04(1.04) | 4.03(1.09) | 0.950 | 3.36(0.93) | 3.42(1.00) | 0.163 | P<0.001 |
| WC (cm) | 96.51(11.34) | 96.17(11.45) | 0.684 | 85.52(11.94) | 87.09(12.81) | 0.001 | P<0.001 |
| SBP (mmHg) | 134.29(22.40) | 139.01(26.37) | 0.019 | 115.06(16.57) | 117.88(19.06) | P<0.001 | P<0.001 |
| DBP (mmHg) | 83.29(10.99) | 83.27(11.59) | 0.886 | 76.41(10.02) | 77.47(10.64) | 0.003 | P<0.001 |
| BMI (kg/m^2^) | 29.84(4.77) | 29.11(5.00) | 0.079 | 26.98(4.68) | 27.39(5.34) | 0.025 | P<0.001 |
| Smoking, No.(%) | 23(3.7) | 6(3.3) | 0.824 | 102(3.5) | 56(5.9) | 0.002 | 0.746 |

|  | | Phase 1 (1999-2002) | | Phase 2 (2002-2005) | | Phase 3 (2005-2008) | | Phase 4 (2008-2011) | | P-value |
| --- | --- | --- | --- | --- | --- | --- | --- | --- | --- | --- |
|  |  | Mean | CI,95% | Mean | CI,95% | Mean | CI,95% | Mean | CI,95% |  |
| FPG (mmol/L) | DM | 8.15 | 7.84-8.45 | 8.39 | 8.04-8.74 | 8.34 | 7.98-8.70 | 8.33 | 7.95-8.71 | 0.740 |
|  | Non-DM | 4.97 | 4.95-4.99 | 5.04 | 5.01-5.06 | 4.99 | 4.97-5.01 | 5.30 | 5.28-5.32 | 0.000 |
| HDL-C (mmol/L) | DM | 0.98 | 0.95-1.00 | 0.91 | 0.89-0.93 | 0.97 | 0.95-1.00 | 1.09 | 1.07-1.12 | 0.000 |
|  | Non-DM | 0.99 | 0.98-1.00 | 0.90 | 0.89-0.91 | 0.98 | 0.97-0.99 | 1.10 | 1.09-1.11 | 0.000 |
| TGs (mmol/L) median(IQR) | DM | 2.79 | 2.59-3.00 | 2.31 | 2.16-1.45 | 2.18 | 2.03-2.34 | 1.85 | 1.74-1.96 | 0.000 |
|  | Non-DM | 1.94 | 1.89-1.99 | 1.87 | 1.81-1.92 | 1.89 | 1.84-1.95 | 1.80 | 1.76-1.85 | 0.002 |
| TC (mmol/L) | DM | 5.74 | 5.63-5.85 | 5.18 | 5.07-5.28 | 4.97 | 4.86-5.08 | 4.70 | 4.59-4.82 | 0.000 |
|  | Non-DM | 5.23 | 5.19-5.28 | 4.93 | 4.88-4.98 | 4.94 | 4.89-4.98 | 4.98 | 4.93-5.02 | 0.000 |
| Non-HDL-C (mmol/L) | DM | 4.76 | 4.15-4.37 | 4.26 | 4.15-4.37 | 3.99 | 3.89-4.10 | 3.60 | 3.49-3.71 | 0.000 |
|  | Non-DM | 4.23 | 4.19-4.28 | 4.02 | 3.98-4.07 | 3.96 | 3.91-4.00 | 3.87 | 3.82-3.91 | 0.000 |
| LDL-C (mmol/L)^a^ | DM | 3.64 | 3.55-3.77 | 3.28 | 3.19-3.36 | 3.10 | 3.01-3.18 | 2.82 | 2.73-2.92 | 0.000 |
|  | Non-DM | 3.36 | 3.33-3.40 | 3.19 | 3.16-3.23 | 3.13 | 3.09-3.16 | 3.07 | 3.03-3.11 | 0.000 |
| WC (cm) | DM | 94.89 | 93.90-95.88 | 98.53 | 97.51-99.55 | 98.56 | 97.46-99.66 | 98.66 | 97.50-99.82 | 0.000 |
|  | Non-DM | 87.42 | 86.96-87.87 | 93.85 | 93.33-94.37 | 95.60 | 95.12-96.08 | 97.28 | 96.78-97.78 | 0.000 |
| WHR | DM | 0.96 | 0.96-0.97 | 0.99 | 0.98-0.99 | 1.00 | 0.99-1.01 | 1.00 | 0.99-1.01 | 0.000 |
|  | Non-DM | 0.91 | 0.90-0.91 | 0.94 | 0.94-0.95 | 0.96 | 0.96-0.97 | 0.97 | 0.96-0.97 | 0.000 |
| SBP (mmHg) | DM | 132.06 | 129.90-134.22 | 130.93 | 128.61-133.24 | 130.58 | 128.11-133.05 | 132.14 | 129.84-134.44 | 0.724 |
|  | Non-DM | 118.49 | 117.78-119.19 | 118.74 | 117.89-119.58 | 118.80 | 117.97-119.63 | 120.06 | 119.26-120.87 | 0.024 |
| DBP (mmHg) | DM | 81.48 | 80.33-82.64 | 78.36 | 77.21-79.52 | 77.91 | 76.69-79.13 | 80.34 | 79.05-81.64 | 0.000 |
|  | Non-DM | 76.89 | 76.45-77.33 | 75.32 | 74.80-75.85 | 75.91 | 75.44-76.37 | 79.45 | 78.94-79.95 | 0.000 |
| BMI (kg/m^2^) | DM | 27.56 | 27.19-27.93 | 27.46 | 27.06-27.87 | 27.51 | 27.09-27.92 | 27.12 | 26.68-27.56 | 0.447 |
|  | Non-DM | 25.48 | 25.32-25.65 | 26.44 | 26.24-26.64 | 26.86 | 26.67-27.05 | 27.25 | 27.05-27.44 | 0.000 |

|  | | Phase 1 (1999-2002) | | Phase 2 (2002-2005) | | Phase 3 (2005-2008) | | Phase 4 (2008-2011) | | P-value |
| --- | --- | --- | --- | --- | --- | --- | --- | --- | --- | --- |
|  |  | Mean | CI,95% | Mean | CI,95% | Mean | CI,95% | Mean | CI,95% |  |
| FPG (mmol/L) | DM | 8.80 | 8.50-9.10 | 8.86 | 8.55-9.18 | 8.62 | 8.33-8.92 | 9.07 | 8.72-9.42 | 0.883 |
|  | Non-DM | 4.86 | 4.84-4.87 | 4.92 | 4.91-4.94 | 4.88 | 4.86-4.90 | 5.18 | 5.16-5.21 | 0.000 |
| HDL-C (mmol/L) | DM | 1.12 | 1.10-1.14 | 1.04 | 1.02-1.06 | 1.11 | 1.09-1.14 | 1.26 | 1.24-1.29 | 0.000 |
|  | Non-DM | 1.18 | 1.16-1.19 | 1.07 | 1.06-1.08 | 1.16 | 1.15-1.17 | 1.32 | 1.31-1.34 | 0.000 |
| TGs (mmol/L) median (IQR) | DM | 2.68 | 2.55-2.80 | 2.56 | 2.43-2.68 | 2.42 | 2.29-2.54 | 2.20 | 2.09-2.31 | 0.000 |
|  | Non-DM | 1.60 | 1.57-1.64 | 1.62 | 1.58-1.66 | 1.58 | 1.54-1.61 | 1.53 | 1.49-1.56 | 0.004 |
| TC (mmol/L) | DM | 6.30 | 6.19-6.41 | 5.74 | 5.63-5.85 | 5.60 | 5.51-5.70 | 5.25 | 5.12-5.36 | 0.000 |
|  | Non-DM | 5.32 | 5.28-5.36 | 5.05 | 5.01-5.09 | 5.03 | 4.99-5.06 | 5.08 | 5.04-5.12 | 0.000 |
| Non-HDL-C (mmol/L) | DM | 5.17 | 5.07-5.28 | 4.70 | 4.59-4.81 | 4.49 | 4.39-4.58 | 3.98 | 3.86-4.10 | 0.000 |
|  | Non-DM | 4.14 | 4.10-1.18 | 3.97 | 3.93-4.02 | 3.86 | 3.82-3.90 | 3.75 | 3.72-3.79 | 0.000 |
| LDL-C (mmol/L)^a^ | DM | 4.04 | 3.96-4.12 | 3.64 | 3.56-3.73 | 3.48 | 3.41-3.56 | 3.07 | 2.10-3.17 | 0.000 |
|  | Non-DM | 3.36 | 3.32-3.39 | 3.20 | 3.16-3.24 | 3.11 | 3.08-3.14 | 3.03 | 2.99-3.06 | 0.000 |
| WC (cm) | DM | 96.51 | 95.60-97.41 | 99.32 | 98.31-100.33 | 98.46 | 97.44-99.48 | 99.88 | 98.82-100.95 | 0.000 |
|  | Non-DM | 85.53 | 85.09-85.97 | 89.88 | 89.37-90.39 | 89.23 | 88.73-89.72 | 94.59 | 94.13-95.05 | 0.000 |
| WHR | DM | 0.90 | 0.90-o.91 | 0.93 | 0.93-0.94 | 0.94 | 0.93-0.94 | 0.99 | 0.91-1.00 | 0.000 |
|  | Non-DM | 0.82 | 0.82-0.82 | 0.85 | 0.85-0.86 | 0.85 | 0.85-0.86 | 0.92 | 0.91-0.92 | 0.000 |
| SBP (mmHg) | DM | 134.31 | 132.52-136.11 | 133.04 | 131.05-135.02 | 130.57 | 128.47-132.66 | 131.51 | 129.34-133.69 | 0.082 |
|  | Non-DM | 115.06 | 114.45-115.67 | 113.27 | 112.52-114.01 | 111.49 | 110.79-112.18 | 114.93 | 114.22-115.63 | 0.000 |
| DBP (mmHg) | DM | 83.21 | 82.34-84.08 | 79.08 | 78.11-80.04 | 76.71 | 75.7-77.68 | 79.05 | 77.95-80.15 | 0.000 |
|  | Non-DM | 76.41 | 76.04-76.78 | 74.22 | 73.79-74.64 | 72.24 | 71.84-72.64 | 75.65 | 75.23-76.07 | 0.000 |
| BMI (kg/m^2^) | DM | 29.84 | 29.46-30.22 | 30.23 | 29.80-30.67 | 30.21 | 29.78-30.64 | 29.99 | 29.52-30.45 | 0.484 |
|  | Non-DM | 26.97 | 26.80-27.14 | 28.37 | 28.17-28.56 | 28.51 | 28.32-28.70 | 29.58 | 29.09-30.06 | 0.000 |

|  | | Phase 1 (1999-2002) | | Phase 2 (2002-2005) | | Phase 3 (2005-2008) | | Phase 4 (2008-2011) | | P for trend | P for interaction |
| --- | --- | --- | --- | --- | --- | --- | --- | --- | --- | --- | --- |
|  |  | Mean | CI,95% | Mean | CI,95% | Mean | CI,95% | Mean | CI,95% |  |  |
| FPG (mmol/L) | DM | 8.23 | 7.97-8.49 | 8.20 | 7.95-8.45 | 8.33 | 8.07-8.59 | 8.71 | 8.41-9.01 | 0.01 | 0.188 |
|  | Non-DM | 4.93 | 4.92-4.95 | 4.95 | 4.94-4.97 | 4.90 | 4.89-4.92 | 5.18 | 5.17-5.20 | P<0.001 |  |
| HDL-C (mmol/L) | DM | 1.08 | 1.05-1.10 | 0.99 | 0.97-1.01 | 1.06 | 1.05-1.21 | 1.19 | 1.17-1.21 | P<0.001 | 0.07 |
|  | Non-DM | 1.11 | 1.10-1.12 | 1.01 | 1.00-1.02 | 1.09 | 1.08-1.10 | 1.23 | 1.22-1.24 | P<0.001 |  |
| TGs (mmol/L)  median(IQR) | DM | 2.69 | 2.54-2.84 | 2.39 | 2.29-2.50 | 2.33 | 2.21-2.44 | 2.13 | 2.02-2.23 | P<0.001 | P<0.001 |
|  | Non-DM | 1.76 | 1.73-1.79 | 1.70 | 1.67-1.73 | 1.68 | 1.66-1.72 | 1.61 | 1.58-1.64 | P<0.001 |  |
| TC (mmol/L) | DM | 5.97 | 5.88-6.06 | 5.46 | 5.37-5.55 | 5.33 | 5.25-5.41 | 5.09 | 4.99-5.19 | P<0.001 | P<0.001 |
|  | Non-DM | 5.36 | 5.33-5.39 | 4.95 | 4.92-4.98 | 4.92 | 4.89-4.95 | 4.93 | 4.90-4.96 | P<0.001 |  |
| Non-HDL-C  (mmol/L) | DM | 4.89 | 4.79-4.98 | 4.47 | 4.38-4.55 | 4.26 | 4.18-4.34 | 3.90 | 3.81-4.00 | P<0.001 | P<0.001 |
|  | Non-DM | 4.24 | 4.21-4.27 | 3.94 | 3.91-3.97 | 3.83 | 3.80-3.85 | 3.69 | 3.66-3.72 | P<0.001 |  |
| LDL-C  (mmol/L)^b^ | DM | 3.79 | 3.72-3.86 | 3.47 | 3.40-3.54 | 3.30 | 3.23-3.37 | 3.02 | 2.94-3.10 | P<0.001 | P<0.001 |
|  | Non-DM | 3.41 | 3.39-3.44 | 3.15 | 3.13-3.18 | 3.06 | 3.03-3.08 | 2.95 | 2.93-2.98 | P<0.001 |  |
| WC(cm) | DM | 95.92 | 95.08-96.76 | 98.54 | 97.72-99.36 | 97.90 | 96.74-98.46 | 98.80 | 97.87-99.73 | P<0.001 | P<0.001 |
|  | Non-DM | 87.24 | 90.68-91.33 | 91.00 | 90.68-91.33 | 91.01 | 90.66-91.32 | 94.22 | 93.88-94.56 | P<0.001 |  |
| WHR | DM | 0.93 | 0.93-0.94 | 0.95 | 0.94-0.95 | 0.96 | 0.94-0.96 | 0.98 | 0.98-0.99 | P<0.001 | 0.001 |
|  | Non-DM | 0.86 | 0.86-0.86 | 0.88 | 0.88-0.89 | 0.89 | 0.89-0.90 | 0.92 | 0.92-0.93 | P<0.001 |  |
| SBP (mmHg) | DM | 133.85 | 132.33-135.37 | 129.49 | 127.99-130.98 | 127.51 | 126.04-128.98 | 128.05 | 126.42-129.68 | P<0.001 | 0.823 |
|  | Non-DM | 118.32 | 117.83-118.81 | 114.48 | 114.02-114.94 | 112.57 | 112.14-113.00 | 113.71 | 113.27-114.16 | P<0.001 |  |
| DBP (mmHg) | DM | 82.01 | 81.20-82.82 | 78.40 | 77.59-79.21 | 77.10 | 76.27-77.91 | 79.77 | 78.85-80.68 | P<0.001 | P<0.001 |
|  | Non-DM | 76.95 | 76.64-77.26 | 74.28 | 73.97-74.59 | 73.22 | 72.93-73.51 | 76.39 | 76.07-76.71 | P<0.001 |  |
| BMI (kg/m^2^) | DM | 28.86 | 28.52-29.52 | 29.2 | 28.95-29.63 | 29.2 | 28.79-29.50 | 29.10 | 28.70-29.49 | 0.295 | P<0.001 |
|  | Non-DM | 26.58 | 26.44-26.72 | 27.45 | 27.32-27.58 | 27.66 | 27.53-27.79 | 28.31 | 28.05-28.58 | P<0.001 |  |

|  | | Phase 1  (1999-2002) | Phase 2  (2002-2005) | Phase 3  (2005-2008) | Phase 4  (2008-2011) | P for trend | P for  interaction |
| --- | --- | --- | --- | --- | --- | --- | --- |
| Reached HDL-C goal (%) | DM | 25.36 | 19.10 | 26.47 | 43.59 | P<0.001 | 0.139 |
|  | Non-DM | 33.29 | 21.63 | 30.16 | 50.56 | P<0.001 |  |
| Reached TGs goal (%) | DM | 27.55 | 31.07 | 35.61 | 43.39 | P<0.001 | P<0.001 |
|  | Non-DM | 57.16 | 59.21 | 60.46 | 64.53 | P<0.001 |  |
| Reached Non-HDL-C goal (%) | DM | 8.20 | 13.83 | 18.48 | 32.22 | P<0.001 | P<0.001 |
|  | Non-DM | 48.75 | 58.14 | 64.22 | 68.63 | P<0.001 |  |
| Reached LDL-C goal (%) ^b^ | DM | 8.13 | 15.25 | 19.16 | 33.63 | P<0.001 | P<0.001 |
|  | Non-DM | 50.34 | 60.95 | 67.11 | 70.82 | P<0.001 |  |
| Reached blood pressure control goal (%) | DM | 56.02 | 66.17 | 68.95 | 65.83 | 0.001 | 0.001 |
|  | Non-DM | 93.50 | 94.13 | 94.18 | 94.46 | 0.07 |  |
| BMI>=30 kg/m^2^ | DM | 38.20 | 41.65 | 39.52 | 40.76 | 0.403 | P<0.001 |
|  | Non-DM | 21.05 | 30.45 | 28.79 | 31.99 | P<0.001 |  |
| Waist>=95 cm | DM | 54.82 | 67.49 | 62.49 | 67.48 | P<0.001 | P<0.001 |
|  | Non-DM | 27.78 | 43.03 | 42..41 | 49.03 | P<0.001 |  |
| Smoking (%) | DM | 9.75 | 13.54 | 11.81 | 12.83 | 0.042 | P<0.001 |
|  | Non-DM | 12.4 | 17.64 | 17.49 | 19.21 | P<0.001 |  |
| Glucose-lowering medication use (%) | DM | 28.65 | 37.14 | 48.95 | 55.98 | P<0.001 | - |
|  | Non-DM | - | - | - | - | - |  |
| Lipid-lowering medication use (%) | DM | 9.59 | 8.40 | 14.52 | 22.93 | P<0.001 | 0.418 |
|  | Non-DM | 1.83 | 1.95 | 2.54 | 4.45 | P<0.001 |  |
| Antihypertensive medication use (%) | DM | 19.23 | 21.68 | 13.91 | 31.18 | P<0.001 | 0.344 |
|  | Non-DM | 4.75 | 5.29 | 2.92 | 6.37 | P<0.001 |  |
